# Establishment and evaluation of a nomogram prediction model for the risk of vascular calcification in stage 5 chronic kidney disease patients

**DOI:** 10.1038/s41598-023-48275-2

**Published:** 2024-01-10

**Authors:** Yan Yang, Wenxue Liang, Wenyu Gong, Shishi Li, Sining Chen, Zhiqian Yang, Chaoying Kuang, Yuzhen Zhong, Demao Yang, Fanna Liu

**Affiliations:** 1 Department of Nephrology, The First Affiliated Hospital of Jinan University, Jinan University, 613 W. Huangpu Avenue, Guangzhou, 510632 Guangdong China; 2 Department of General Practice, Puning People’s Hospital, Puning, 515300 Guangdong China

**Keywords:** Diseases, Nephrology, Risk factors

## Abstract

Vascular calcification (VC) is a common complication of chronic kidney disease (CKD) that has a detrimental effect on patients' survival and prognosis. The aim of this study was to develop and validate a practical and reliable prediction model for VC in CKD5 patients. The medical records of 544 CKD5 patients were reviewed retrospectively. Multivariate logistic regression analysis was used to identify the independent risk factors for vascular calcification in patients with CKD5 and then created a nomogram prediction model. The area under the receiver operating characteristic curve (AUC), Hosmer–Lemeshow test, and decision curve analysis (DCA) were used to assess model performance. The patients were split into groups with normal and high serum uric acid levels, and the factors influencing these levels were investigated. Age, BUN, SUA, P and TG were independent risk factors for vascular calcification in CKD5 patients in the modeling group (*P* < 0.05). In the internal validation, the results of model showed that the AUC was 0.917. No significant divergence between the predicted probability of the nomogram and the actual incidence rate (x^2^ = 5.406, *P* = 0.753) was revealed by the calibration plot and HL test, thus confirming that the calibration was satisfactory. The external validation also showed good discrimination (AUC = 0.973). The calibration chart and HL test also demonstrated good consistency. Besides, the correlation analysis of serum uric acid levels in all CKD5 patients revealed that elevated uric acid levels may be related to gender, BUN, P, and TG.

## Introduction

Chronic kidney disease (CKD) is a multifactorial disease with persistent disorders in renal structure and/or function. Early renal protection measures are needed to delay the progression to end stage renal disease ESRD. It is estimated that about 13.4% of the global population suffers from CKD, and the incidence of CKD is high in China (10.8%)^1^. The burden of CKD is not limited to its impact on the need for renal replacement therapy, but also increases cardiovascular risk in the affected population, directly affecting the global burden of death from cardiovascular disease. Chronic kidney disease-mineral and bone disorder (CKD-MBD) can cause a variety of symptoms such as hypocalcemia, hyperphosphatemia, secondary hyperparathyroidism, vitamin D deficiency, and vascular calcification^2^. These symptoms can have a major influence on the quality of life of CKD patients^3^. Vascular calcification (VC) is an active pathological process in which minerals in the form of hydroxyapatite are deposited ectopic on the wall of blood vessels and is characterized by vascular smooth muscle cells (VSMCS)^4^. In addition to raising the risk of hospitalization, cardiovascular disease events and mortality, and all-cause mortality in patients with non-dialysis dependent chronic kidney disease, VC is an independent incremental predictor of all-cause mortality in dialysis dependent patients with end-stage renal disease^5,6^.

However, VC is an asymptomatic disease that can develop over years without any signs or symptoms, early diagnosis of the condition can be a significant clinical challenge. Conventional X-ray examination can detect extra-skeletal calcifications and is less expensive and radiation-intensive. Studies have shown that lateral dual-energy X-ray absorptiometry spine imaging can be used to determine the abdominal aortic calcification score (AAC)^7^. The gold standard for assessing coronary artery, aorta, and valve calcification is the CT scan, which can more precisely understand calcification by examining the volume and density of coronary artery calcification^8,9^. Inadequately, it lacks sensitivity to distinguish between intimal and media layer calcifications and has limitations in detecting microcalcifications^10^. Guidelines currently recommend lateral abdominal X-ray examination for the evaluation of VC in CKD3-5 patients^11^. There are still restrictions in the early diagnosis of VC by imaging examination because of the complicated pathological mechanism of VC.

Several pathways connect vascular calcification to CKD, but the mechanism is unknown. VC in CKD patients is usually affected by multiple factors, including common traditional factors such as age, obesity, smoking, family history, hypertension, dyslipidemia, and hyperglycemia^12–14^. With the progress of in-depth research, more and more non-traditional risk factors have been paid attention to, including inflammation, mineral metabolism disorders, oxidative stress, endothelial dysfunction, malnutrition, bone diseases, anemia, etc., all of which affect the occurrence and development of VC from different ways^15–17^.

There is currently no reliable and robust predictive model that can be used to identify high-risk patients and prevent VC deterioration. The majority of clinical event prediction models have extensively used nomogram modeling, which is a trustworthy statistical tool. It is a visual line chart that combines various clinical indicators with multivariate regression analysis to predict the likelihood of a particular clinical outcome^18,19^. In order to create a nomogram to quantitatively assess the risk of vascular calcification in CKD5 patients, this study performed a retrospective analysis of CKD5 patients based on accumulated data in the hospital information system.

## Methods

### Patients

The data of 634 patients were collected from the information and data system of the First Affiliated Hospital of Jinan University and the hemodialysis center of Dongpu Hospital. Inclusion criteria were as follows: patients that met the KDIGO2012 expert consensus CKD5 diagnostic criteria; patients that met lateral abdominal X-ray results within the previous year. Exclusion criteria include: patients under the age of 18; patients with acute or chronic infectious diseases, acute exacerbation of cardiovascular or cerebrovascular diseases, acute gout, autoimmune systemic diseases, primary hyperparathyroidism, hematological diseases, or malignant tumors; patients who are pregnant or lactating.

The lateral abdominal plain films were taken by experienced radiologists who were not aware of the patient information. According to the calcification of the abdominal aorta, patients were split into non-calcification and calcification groups.

**Figure 1:**
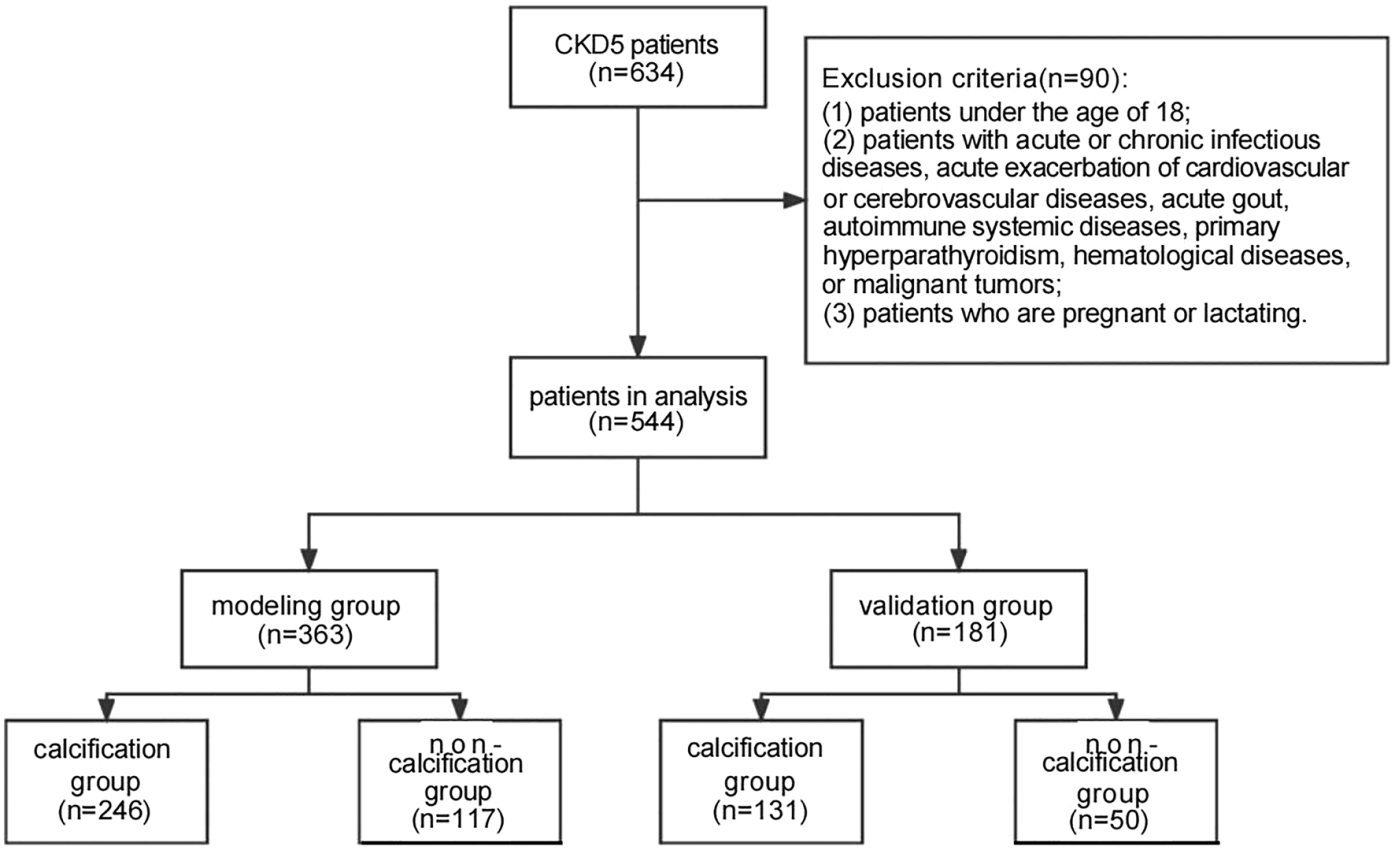
Flowchart depicting the inclusion of CKD5 patients in this study.

This study included 544 patients, with 363 patients in the modeling group and 181 patients in the validation group (Fig. 1). All patients provided written informed consent to ensure the accuracy of the investigation.

### Data collection

The personal data of the patients were collected through the hospital information system platform, including age, gender, height, weight, body mass index (BMI), resting blood pressure, history of underlying diseases (hypertension, diabetes, and cardiovascular disease), smoking history, and drinking history. All patients had fasting venous blood drawn. Hemoglobin (HGB), serum albumin (ALB), blood urea nitrogen (BUN), serum creatinine (Scr), serum uric acid (SUA), calcium, phosphorus, alkaline phosphatase (ALP), total parathyroid hormone (iPTH), triglyceride (TG), total cholesterol (TC), high density lipoprotein cholesterol (HDL-C), low density lipoprotein cholesterol (LDL-C) levels were measured. Radiologists evaluated and scored lateral abdominal X-ray plain film to detect vascular calcification.

### Statistical analyses

R (version 4.0.3 https://www.R-project.org) software and SPSS (version 26.0 http://www.spss.com.cn) was used for statistical analysis, and measurement data were expressed by mean ± SD. The independent sample t test was used to compare two groups that followed normal distribution, while the rank sum test was used to compare two groups that were disobedient to normal distribution or had uneven variance. Count data were expressed as the number of cases (%), and Pearson chi-square test was used for statistical analysis between the two groups. In order to identify independent risk factors for vascular calcification in CKD5 patients, binary multivariate logistic regression analysis was used. The odds ratio (OR) and 95% confidence interval (CI) were computed. Then we selected factors to create a nomogram to forecast the likelihood of vascular calcification in patients with CKD5 patients. The data from the modeling and validation cohorts underwent internal and external validation to ensure reliability, and the model was assessed using the calibration curve, the Hosmer–Lemeshow (HL) fit curve, and the area under the ROC curve (AUC).

### Ethics statement

This study had been approved by the Institutional Ethics Committee of the First Affiliated Hospital of Jinan University and was in accordance with the Declaration of Helsinki (KY-2021-017). We fully explained the purpose and experimental procedures to each patient. They all volunteered to participate in the program and signed the informed consent.

## Results

### The baseline data and clinical indicators of patients in the modeling group

The clinical information of the patients was summarized in Supplementary Table S1. The modeling group included 363 patients, with 117 in the non-calcification group and 246 in the calcification group. The non-calcification group included 63 females (53.8%) and 54 males (46.2%), while the calcification group included 105 females (42.7%) and 141 males (57.3%). Gender differences between the two groups were statistically significant (*P* = 0.046, *P* < 0.05). The non-calcification group had a median age of 48 years, while the calcification group had a median age of 64 years, and the difference was statistically significant (*P* < 0.001). Patients with renal replacement therapy were present in both groups. Hypertension, coronary heart disease and cerebral infarction were the main complications in both groups. Other characteristics including BMI, smoking history, and drinking history were not significantly different between the two groups. The primary cause of CKD in this study was chronic glomerulonephritis, followed by diabetic nephropathy and hypertensive nephropathy (Supplementary Fig. 1). SBP, DBP, HGB, ALB, eGFR, BUN, Scr, SUA, Ca, P, iPTH, TG, TC, HDL-C, and LDL-C were significantly different between the two groups (*P* < 0.05). There was no significant difference in ALP between the two groups (*P* > 0.05).

### Analysis of risk factors for vascular calcification in the modeling group

In the modeling group, vascular calcification (occurrence = 1, no occurrence = 0) was set as the dependent variable in CKD5 patients. Age, diabetes history, blood pressure, BUN, Scr, eGFR, SUA, Ca, P, ALB, TC, TG, LDL-C, HDL-C, iPTH, and HGB were the factors that significantly differed between the non-calcification group and the calcification group and were therefore set as independent variables. Age, BUN, SUA, P, and TG were independent risk factors for vascular calcification in CKD5 patients, according to multivariate logistic regression analysis (*P* < 0.05 Table 1).

**Table 1:** Multivariate Logistic regression analysis of the risk factors of vascular calcification in patients with CKD5 patients.

Dependent	B	SE	Z	*P*-value	OR (95% CI)
Age (year)	0.090	0.013	7.111	< 0.001	1.09 [1.07, 1.12]
BUN (mmol/L)	0.080	0.019	4.288	< 0.001	1.08 [1.04, 1.12]
SUA (µmol/L)	0.006	0.002	3.439	0.001	1.01 [1, 1.01]
P (mmol/L)	3.992	0.638	6.254	< 0.001	54.15 [15.5, 189.2]
TG (mmol/L)	− 0.262	0.130	-2.013	0.044	0.77 [0.6, 0.99]

### Construction of a nomogram prediction model for vascular calcification in CKD5 patients

**Figure 2:**
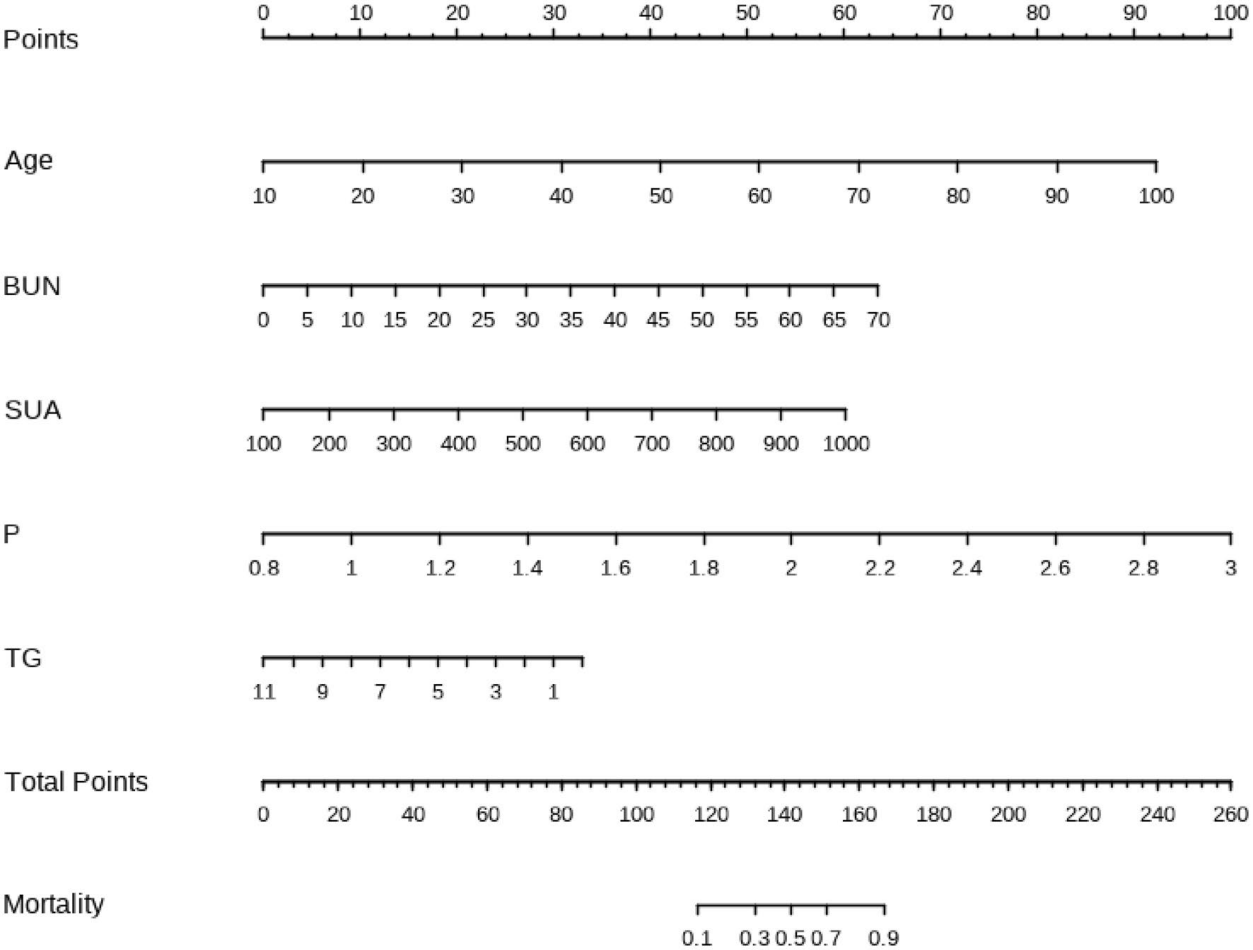
Nomogram prediction model for the occurrence of vascular calcification in CKD5 patients.

Age, BUN, SUA, P and TG of patients in the modeling group were included in the prediction model to construct a nomogram to predict the occurrence of vascular calcification in CKD5 patients (Fig. 2). Each predictor variable received a score in the multiple logistic regression analysis proportionally based on its OR.

### Internal validation of the nomogram prediction model in the modeling group

**Figure 3:**
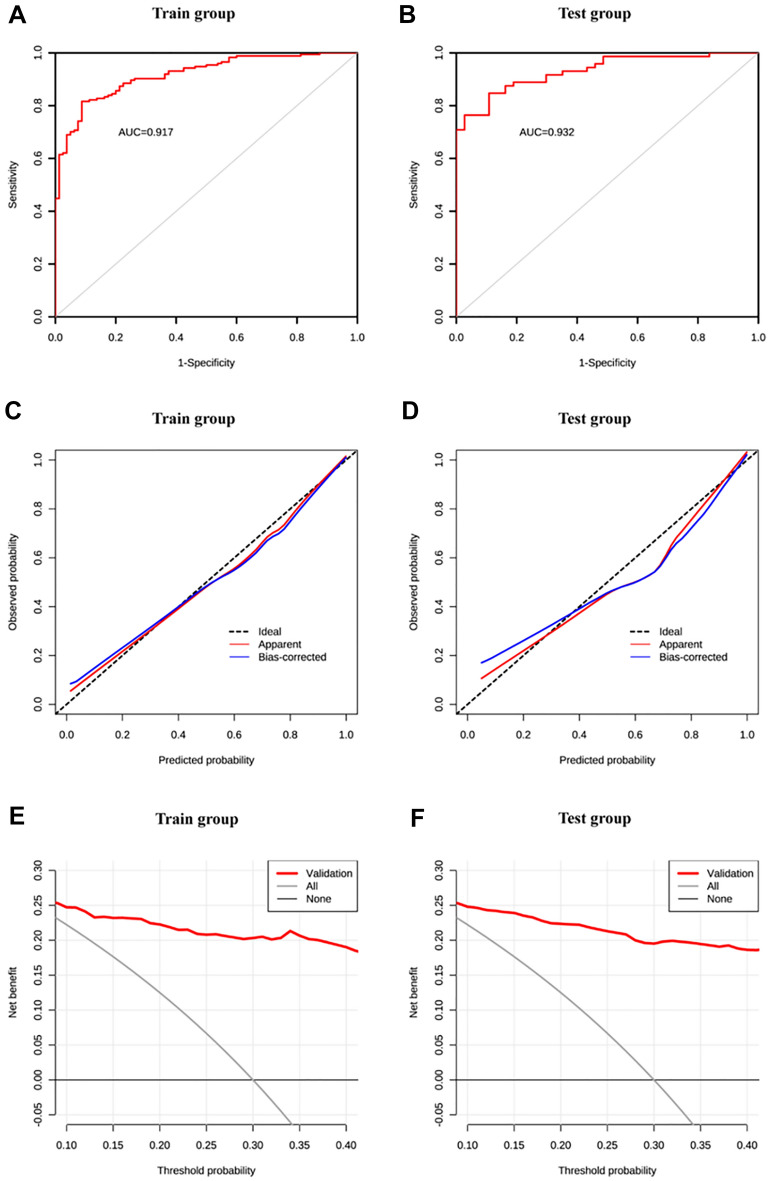
Internal validation of the prediction model (**A**) AUC of the training set. (**B**) AUC of validation set. (**C**) calibration curve of training set. (**D**) Calibration curve of validation set. (**E**) Decision curve of the test set. (**F**) Decision curve of validation set.

The dataset of the modeling group was randomly assigned to the training set and the validation set at a ratio of 7:3, with a sample size of 254 for the training set and 109 for the validation set. The results of ROC curve showed that the AUC of the training set was 0.917, and the AUC of the validation set was 0.932, indicating that the model had good discrimination (Fig. 3A,B). The calibration curve revealed no significant difference between the probability predicted by the nomogram and the actual incidence (χ^2^ = 5.406, *P* = 0.753), which indicated good calibration (Fig. 3C,D). The clinical viability of the prediction model was assessed using DCA. The net benefit of patients was greater than the two extreme curves in the figure when the threshold risk probability was between 1 and 99%, demonstrating that this range had a high clinical treatment efficiency (Figs. 3E,F).

### External validation of the nomogram prediction model in the validation group

**Figure 4:**
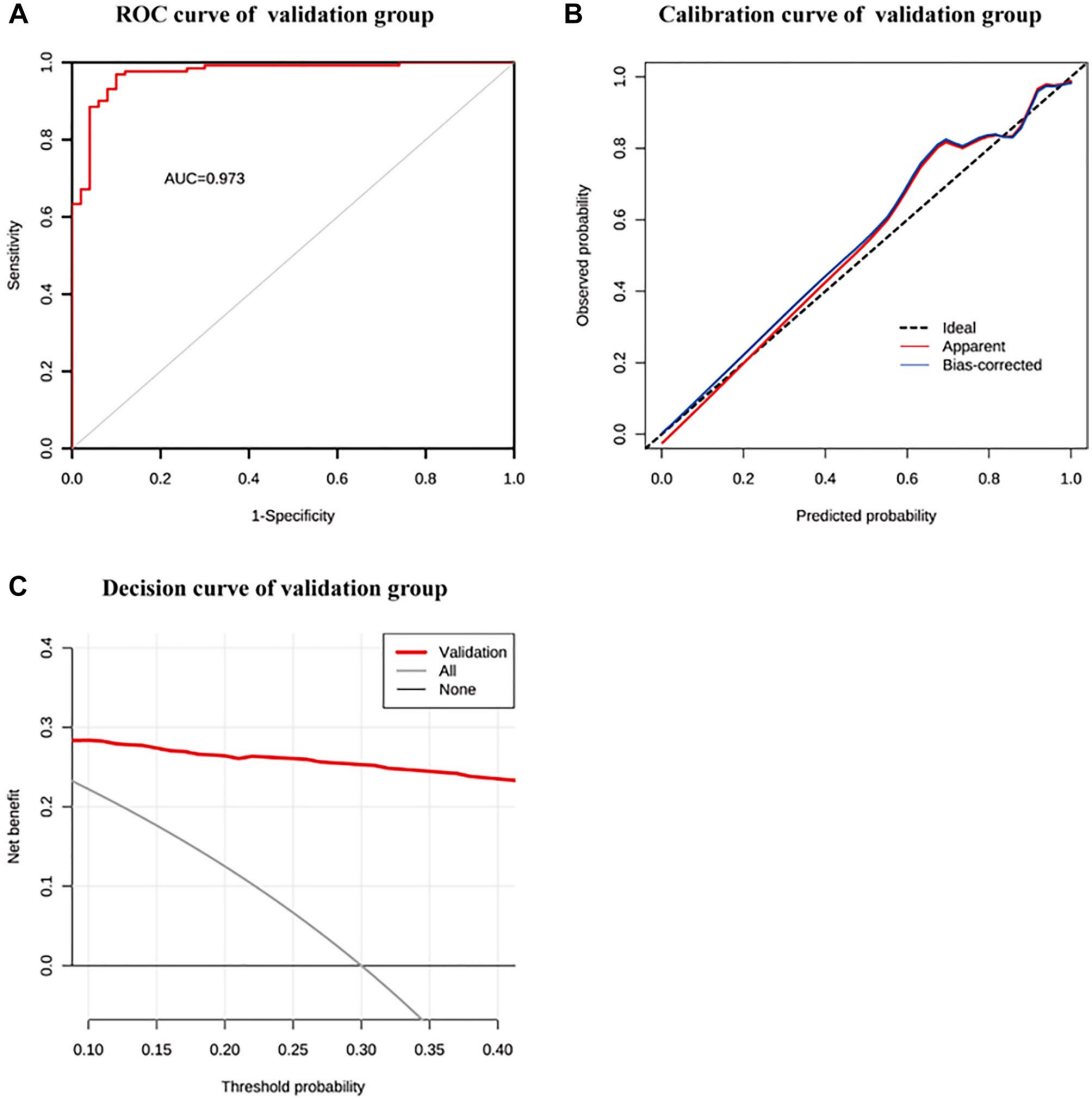
External validation of the prediction model (**A**) AUC of the external validation set. (**B**) Calibration curve of external validation set. (**C**) Decision curve of the external validation set.

The validation group consisted of 181 patients in total, including 105 males and 76 females (Supplementary Table S2). These patients were also divided into non-calcification group (n = 50) and calcification group (n = 131). There were significant differences in Age, BUN, SUA, P and TG between the two groups (*P* < 0.05 Table 2). AUC was 0.973 when patients were included in the external validation model, indicating that the model had good discrimination power (Fig. 4A,B). The calibration curve demonstrated good calibration because the probability predicted by the nomogram was nearly identical to the actual incidence rate (Fig. 4C).

**Table 2:** Comparison of baseline between the non-calcification group and calcification group in the validation group.

Dependent	Non-calcification group (n = 50)	Calcification group (n = 131)	t	*P*-value
Age (year)	48.70 ± 12.74	60.38 ± 14.00	− 5.143	< 0.001
BUN (mmol/L)	20.22 ± 3.99	23.87 ± 4.48	− 5.052	< 0.001
SUA (µmol/L)	442.79 ± 79.28	480.77 ± 108.92	− 2.583	< 0.001
P (mmol/L)	1.17 ± 0.11	1.44 ± 0.11	− 14.982	< 0.001
TG (mmol/L)	1.94 ± 0.64	1.73 ± 0.47	2.097	0.040

### Correlation analysis of serum uric acid levels in CKD5 patients

**Figure 5:**
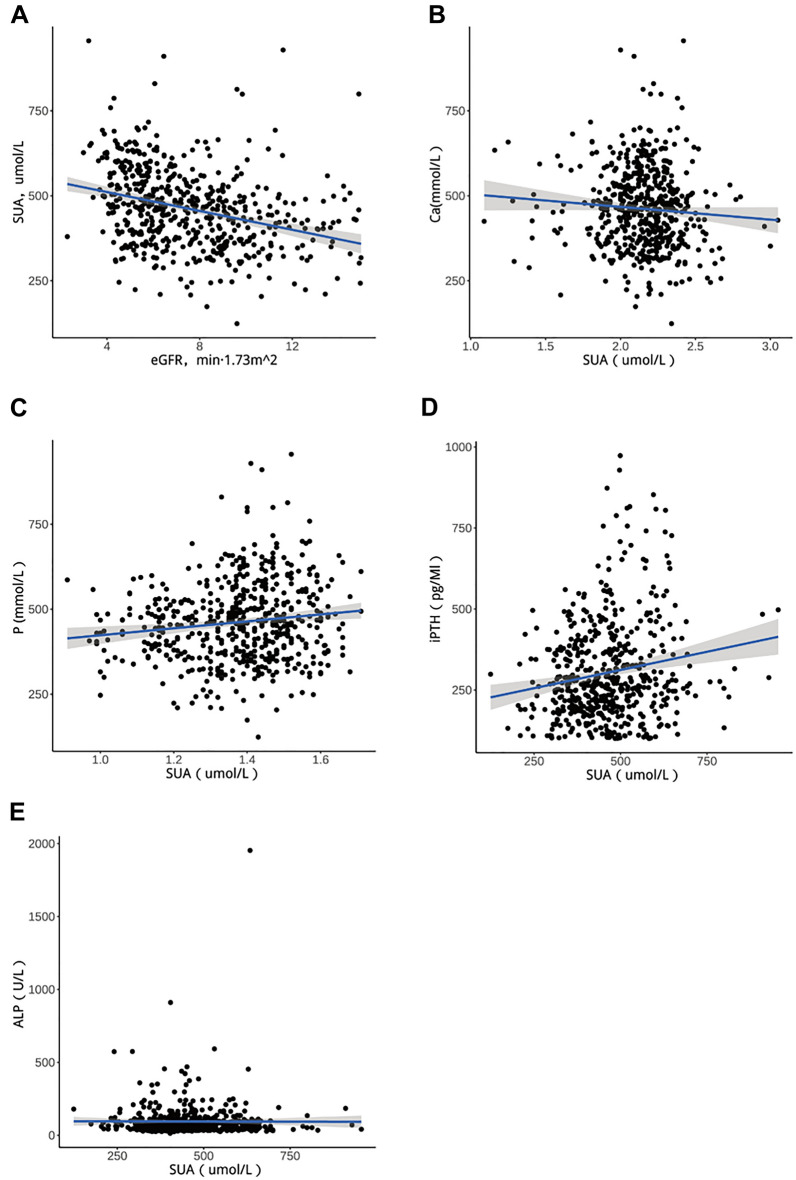
Correlation analysis of serum uric acid levels in CKD5 patients (**A**) Linear plot of SUA level and eGFR. (**B**) Linear plot of SUA level and Ca. (**C**) Linear plot of SUA level and P. (**D**) Linear plot of SUA level and iPTH. (**E**) Linear plot of SUA level and ALP.

The results of this study showed that the serum uric acid level was negatively correlated with eGFR (r = − 0.312, *P* < 0.05 Fig. 5A). Based on Chinese guidelines, hyperuricemia was defined as fasting serum uric acid levels > 420 μmol/L (7 mg/dL) in men and > 360 μmol/L (6 mg/dL) in women on two different days while on a normal purine diet. Then the patients were divided into normal uric acid group (n = 167) and hyper uric acid group (n = 377). SBP, DBP, BUN, SCr, eGFR, SUA, calcium, phosphorus, ALB, TC, TG, LDL-C, HDL-C, iPTH, and HGB were all significantly different between the two groups (*P* < 0.05 Supplementary Table S3). There was no significant difference in ALP between the two groups (*P* > 0.05). Gender, BUN, P and TG were independent risk factors for elevated serum uric acid levels in CKD5 patients (*P* < 0.05, Table 3). The correlation analysis between serum uric acid and abnormal mineral metabolism in CKD5 patients showed that P and iPTH were positively correlated with serum uric acid, and the difference was statistically significant. There was no significant correlation between Ca, ALP and serum uric acid levels (Table 4, Fig. 5B–E).

**Table 3:** Multivariate logistic regression analysis of the risk factors of elevated SUA level in patients with CKD5.

Dependent	B	SE	Z	*P*-value
Gender	0.998	0.274	3.641	< 0.001
BUN (mmol/L)	0.088	0.018	4.784	< 0.001
P (mmol/L)	2.003	0.774	2.589	0.010
TG (mg/dL)	− 0.236	0.110	-2.152	0.031

**Table 4:** Correlation analysis of SUA level and abnormal mineral metabolism in patients with CKD5.

Dependent	r	*P*-value
P (mmol/L)	0.135	0.002
Ca (mmol/L)	− 0.007	0.073
iPTH (pg/mL)	0.173	0.000
ALP (U/L)	− 0.004	0.922

## Discussion

CKD is associated with increased morbidity and mortality of cardiovascular disease, and its poor prognostic outcome is also associated with CKD-MBD. According to studies, endothelial dysfunction, atrial stiffness, and vascular calcification are all significantly influenced by CKD-MBD^20^. In this study, Age, BUN, SUA, P, and TG were identified as independent risk factors for vascular calcification in patients with CKD5. We developed a nomogram based on these five factors and verified the reliability of the nomogram model. In the validation cohort, the AUC was 0.917, while it was 0.973 in the training cohort. The calibration chart and HL test also demonstrated good consistency, suggesting that the model had good discrimination and accuracy.

It has been identified in the general population, CVD population, and CKD population that age is an independent risk factor for VC. The results of the present study similarly showed that the age of the calcification group was higher than that of the non-calcification group, and age was positively correlated with VC. The aging process accumulates numerous vascular injury-causing factors. There is a large amount of lipid deposition in the intima of the vascular wall, an increase in connective tissue components, and a decrease in smooth muscle and elastin^21^. Age-related increases in vascular calcification risk were observed in CKD patients, and the incidence can rise by 30% between 20 and 90 years of age^22^. Meta-analysis of the factors influencing coronary vessel calcification in patients with chronic kidney disease and end-stage renal disease found that age is a determinant of VC^23^. Early detection and prevention of vascular calcification in elderly CKD patients is critical for improving clinical outcomes.

Dyslipidemia in CKD patients, including high TC and LDL-C, and to a lesser extent TG, always develops in parallel with decreased renal function, even at an early stage^24^. This study found that the calcification group had higher TG levels and lower HDL-C levels compared with the non-calcification group. According to the findings of a Chinese cohort study, a high TG/HDL-C ratio is associated with a higher risk of reduced renal function in the Chinese population^25^. Elevated TG levels speed up lipid exchange, which may result in higher LDL and lower HDL levels^26^. When LDL is oxidized, it produces oxidized low density lipoprotein (ox-LDL), which can induce vascular smooth muscle cells to transdifferentiate into osteoblasts and speed up the progression of VC^27^. Niu et al. evaluated the risk of arterial vascular calcification in CKD patients on maintenance hemodialysis, and the results suggested that higher baseline serum TG level was an independent risk factor for VC^28^.

P, Ca, and iPTH were significantly different in CKD5 patients with vascular calcification in this study. P and iPTH was higher while Ca was lower in the calcification group. However, due to the small number of experimental samples and the error in the detection of experimental indicators, Ca and iPTH could not be used as independent risk factors for VC in this study. Patients may have taken calcium-based phosphate binders, calcitriol, and other drugs that may have had an effect on calcium and phosphorus metabolism as well as iPTH in patients, thereby affecting the experimental results. Elevated levels of calcium and phosphorus motivates VSMCS to differentiate into osteogenic or chondrogenic cells, induces cell death and vesicle release, aggregates phosphate and crystalline calcium during vascular mineralization, and speeds up calcification^29,30^. According to studies, coronary artery calcification was associated with hyperphosphatemia in CKD patients, and elevated phosphate levels (each 0.5 mg/dL increase) were also associated with an increased risk of myocardial infarction and death in CKD patients^31^. Vascular smooth muscle cell death brought on by calcium and phosphate-induced apoptosis was also closely related to vascular calcification in CKD vessels^32^. Studies have shown that iPTH can enhance cartilage matrix expression and intracellular calcium levels, thereby facilitating the development of VC. VC can be caused by both high and low iPTH levels^33^. As previously stated, vascular calcification is an active and controllable process, and metabolic calcium and phosphorus deposition can cause calcification changes. However, because of Ca, P, and iPTH levels measured in this study can only reflect the metabolic level at a specific point in time, they cannot accurately and dynamically assess the long-term evolution of VC. As a result, the relationship between Ca, iPTH, and VC requires further investigation using a large number of clinical data and experiments.

Numerous epidemiological, experimental, and clinical studies have demonstrated that hyperuricemia has a gradually rising prevalence in CKD patients and is also significantly linked to CVD, vascular inflammatory diseases, and mortality^34,35^. Uric acid is the byproduct of the xanthine oxidase-produced purine compound metabolism, and elevated uric acid levels can result from both excessive urate production and decreased renal uric acid excretion^36^. It is still debatable, though, whether uric acid contributes to vascular calcification in the CKD population.

This study further analyzed the factors affecting uric acid. The results revealed that, in patients with CKD5, gender, BUN, P, and TG were independent risk factors for elevated serum uric acid levels. Previous epidemiological and mechanistic studies have verified that a number of variables, including genetics, gender, age, diet, diabetes, and hypertension, have an impact on SUA levels^37^. According to a meta-analysis, male patients with hyperuricemia in mainland China had an average serum uric acid level that was higher than that of female patients, and the gender prevalence was also significantly different, with male patients having a prevalence of 19.4% and female patients having a prevalence of 7.9%^38^. Estrogen may act as a protective factor in HUA and regulate the expression or activity of UA transporters^39^. Elevated TG levels may result in an increase in free fatty acids and a faster breakdown of adenosine triphosphate, both of which lead to an increase in uric acid^40^. Xu et al. found that high TG and low HDL-C were significantly and independently associated with the risk of HUA in a 6-year prospective study of Chinese older adults over 60 years old, and that there was a nonlinear increasing trend among TG, HDL-C, and SUA^41^. Hou et al. found that the risk of HUA in patients with high TG was 2.353 times that in patients with normal TG. After adjusting for confounding factors, TG was still an independent risk factor for HUA, and the risk of HUA increased with the increase of TG level^42^. Besides, the study also discovered a positive relationship between BUN and HUA. SUA levels were higher in patients with decreased eGFR, and BUN was excreted by the kidney. When SUA levels rose, renal metabolic function suffers, and BUN rose as well.

In CKD5 patients, P and iPTH were found to positively correlate with SUA levels, while Ca and ALP did not significantly correlate with SUA levels. P is a separate risk element for HUA. SUA is correlated with serum Ca and P levels, and most patients with CKD5 have problems with calcium and phosphorus metabolism. A Chinese study suggested that SUA was positively correlated with P, iPTH and ALP, and negatively correlated with eGFR in CKD stage 3–5 non-dialysis patients (*P* < 0. 05).

There are several limitations to this study. Firstly, bias in the data may emerge from its collection and evaluation from several centers, which may have an impact on the results than follow. Owing to the demographic characteristics of the sample, our conclusions cannot be generalized to other ethnic groups. Secondly, long-term and dynamic follow-up observation has not been carried out. Furthermore, patient medications such as the use of calcium-containing P-binders, calcitriol, urate-lowering medications, and other medications were not included in this study. The potential impact of medications that interfere with uric acid metabolism on the current findings, including any potential causal connection between SUA levels and VC, could not be completely ruled out by this study.

## Conclusion

Age, BUN, SUA, P and TG have good predictive value for the risk of vascular calcification in CKD5 patients, and the predictive model constructed by this method is helpful for clinical decision-making. In addition, this study observed that high uric acid levels can affect vascular calcification by promoting the disorder of P and iPTH metabolism. In conclusion, the foregoing elements must be taken into account as a whole in order to prevent or treat vascular calcification.

### Supplementary Information

Supplementary Figures.

Supplementary Tables.

Supplementary Information 1.

## Data Availability

The data underlying this article are available in the article and in its online supplementary material.

## Abbreviations

CKD: Chronic kidney disease
CVD: Cardiovascular disease
CKD-MBD: Chronic kidney disease–mineral and bone disorder
VC: Vascular calcification
VSMC: Vascular smooth muscle cell
HUA: Hyperuricemia
KDIGO: Kidney disease improving global outcomes
BMI: Body mass index (Kg/m^2^)
HGB: Hemoglobin
ALB: Albumin
BUN: Blood urea nitrogen
Scr: Serum creatinine
SUA: Serum uric acid
ALP: Alkaline phosphatase
Ipth: Intact parathyroid hormone
Ca: Calcium
P: Phosphorus
TG: Triglyceride
TC: Total cholesterol
LDL-C: Low-density lipoprotein cholesterol
HDL-C: High-density lipoprotein cholesterol
HL: Hosmer lemeshow
ESRD: End stage renal disease
