# Supplementary material for: Establishment and evaluation of a nomogram prediction model for the risk of vascular calcification in stage 5 chronic kidney disease patients
Source: Sci Rep. 2024 Jan 10;14:1025. doi: 10.1038/s41598-023-48275-2 (PMC10781805; doi:10.1038/s41598-023-48275-2)
Supplement: Supplementary file 1 — Supplementary Figures. [file 41598_2023_48275_MOESM1_ESM.docx]

**Title page:**

**Title:** Establishment and evaluation of a nomogram prediction model for the risk of vascular calcification in stage 5 chronic kidney disease patients

**Authors:**

Yan Yang^1*^, Wenxue Liang^2*^ ,Wenyu Gong^2^, Shishi Li^2^, Sining Chen^2^, Zhiqian Yang^2^, Chaoying Kuang^2^,  Yuzhen Zhong^2^,Demao Yang^1#^, Fanna Liu^2#^

* Yan Yang and Wenxue Liang have contributed equally to this work.

1Department of General practice, Puning People’s Hospital, Puning, Guangdong 515300, China

2 Department of Nephrology, The First Affiliated Hospital of Jinan University, Jinan University, Guangzhou, China.

**Corresponding author**:

Demao Yang, Department of General practice, Puning People’s Hospital, Puning, Guangdong 515300, China **E-mail:** Yangdemao2020@163.com

Fanna Liu, Department of Nephrology, The First Affiliated Hospital of Jinan University, 613 W. Huangpu Avenue, Guangzhou, Guangdong, China. **E-mail:** [tliufana@jnu.edu.cn](mailto:tliufana@jnu.edu.cn)

**Supplementary Figure 1**

Primary disease distribution in patients without calcification and in patients with calcification
