# Supplementary material for: Establishment and evaluation of a nomogram prediction model for the risk of vascular calcification in stage 5 chronic kidney disease patients
Source: Sci Rep. 2024 Jan 10;14:1025. doi: 10.1038/s41598-023-48275-2 (PMC10781805; doi:10.1038/s41598-023-48275-2)
Supplement: Supplementary file 2 — Supplementary Tables. [file 41598_2023_48275_MOESM2_ESM.docx]

**Title page:**

**Title:** Establishment and evaluation of a nomogram prediction model for the risk of vascular calcification in stage 5 chronic kidney disease patients

**Authors:**

Yan Yang^1*^, Wenxue Liang^2*^ ,Wenyu Gong^2^, Shishi Li^2^, Sining Chen^2^, Zhiqian Yang^2^, Chaoying Kuang^2^,  Yuzhen Zhong^2^,Demao Yang^1#^, Fanna Liu^2#^

* Yan Yang and Wenxue Liang have contributed equally to this work.

**Affiliations**:

1Department of General practice, Puning People’s Hospital, Puning, Guangdong 515300, China

2 Department of Nephrology, The First Affiliated Hospital of Jinan University, Jinan University, Guangzhou, China.

**Corresponding author**:

Demao Yang, Department of General practice, Puning People’s Hospital, Puning, Guangdong 515300, China **E-mail:** Yangdemao2020@163.com

Fanna Liu, Department of Nephrology, The First Affiliated Hospital of Jinan University, 613 W. Huangpu Avenue, Guangzhou, Guangdong, China. **E-mail:** tliufana@jnu.edu.cn

Supplementary Table S1 Baseline characteristics of non-calcification and calcification groups

| Characteristics | non-calcification group  (n=117) | calcification group  (n=246) | t | *P*-value |
| --- | --- | --- | --- | --- |
| Age | 48.08±14.16 | 63.83±13.71 | -10.122 | <0.001 |
| Gender |  |  | 3.974 | 0.046 |
| Female | 63(53.8%) | 105(42.7%) |  |  |
| Male | 54(46.2%) | 141(57.3%) |  |  |
| Smoking |  |  | 0.526 | 0.468 |
| No | 99(84.6%) | 215(87.4%) |  |  |
| Yes | 18(15.4%) | 31(12.6%) |  |  |
| Drinking |  |  | 0.833 | 0.361 |
| No | 111(94.9%) | 227(92.3%) |  |  |
| Yes | 6(5.1%) | 19(7.7%) |  |  |
| Diabetic |  |  | 19.096 | <0.001 |
| No | 92(78.6%) | 135(54.9%) |  |  |
| Yes | 25(21.4%) | 111(45.1%) |  |  |
| renal replacement therapy |  |  | 26.061 | <0.001 |
| No | 69(58.9%) | 76(30.9%) |  |  |
| Yes | 48(41.1%) | 170(69.1%) |  |  |
| BMI, kg/m^2^ | 23.04±4.48 | 22.96±3.58 | 0.168 | 0.867 |
| SBP, mmHg | 138.45±25.25 | 145.99±26.25 | -2.589 | 0.010 |
| DBP, mmHg | 83.32±16.09 | 78.29±14.37 | 3.000 | 0.003 |
| Scr, mg/dl | 7.94±2.11 | 8.98±2.80 | -3.943 | <0.001 |
| BUN, mmol/L | 14.89±8.64 | 21.85±9.75 | -6.581 | <0.001 |
| eGFR，min·1.73m^2 | 8.54±2.62 | 7.19±2.64 | 4.543 | <0.001 |
| SUA，umol/L | 398.01±83.86 | 486.21±128.71 | -7.813 | <0.001 |
| P, mmol/L | 1.26±0.16 | 1.44±0.10 | -11.669 | <0.001 |
| Ca, mmol/L | 2.23±0.25 | 2.14±0.25 | 3.263 | 0.001 |
| iPTH，pg/Ml | 261.30±110.15 | 327.34±164.72 | -4.515 | <0.001 |
| ALP, U/L | 94.45±107.99 | 102.24±144.63 | -0.573 | 0.567 |
| TC, mmol/L | 4.93±1.63 | 4.46±1.08 | 2.864 | 0.005 |
| TG，mmol/L | 2.05±1.62 | 1.71±1.01 | 2.051 | 0.042 |
| HDL-C，mmol/L | 1.13±0.36 | 1.03±0.32 | 2.688 | 0.008 |
| LDL-C，mmol/L | 2.74±1.13 | 2.45±0.83 | 2.515 | 0.013 |
| HGB, g/dl | 119.19±22.79 | 102.08±21.71 | 6.905 | <0.001 |
| ALB，g/L | 39.26±6.47 | 36.69±6.49 | 3.510 | <0.001 |
| Complication |  |  | 29.544 | <0.001 |
| Hypertension | 46(39.3%) | 126(51.2%) |  |  |
| Coronary heart disease | 15(12.8%) | 52(21.1%) |  |  |
| Cerebral infarction | 5(4.3%) | 25(10.2%) |  |  |

Supplementary Table S2 Baseline characteristics of non-calcification and calcification groups in the validation group

| Characteristics | non-calcification group  (n=50) | calcification group  (n=131) | t | *P*-value |
| --- | --- | --- | --- | --- |
| Age | 48.70±14.16 | 60.38±14.1 | -5.143 | <0.001 |
| Gender |  |  | 0.451 | 0.502 |
| Female | 31(62.0%) | 74(56.5%) |  |  |
| Male | 19(38.0%) | 57(43.5%) |  |  |
| Smoking |  |  | 10.026 | 0.200 |
| No | 29(58.0%) | 106(80.9%) |  |  |
| Yes | 21(42.0%) | 25(19.1%) |  |  |
| Drinking |  |  | 0.675 | 0.411 |
| No | 42(84.0%) | 116(88.5%) |  |  |
| Yes | 8(16.0%) | 15(11.45%) |  |  |
| Diabetic |  |  | 1.151 | 0.283 |
| No | 33(66.0%) | 75(57.3%) |  |  |
| Yes | 17(34.0%) | 56(42.7%) |  |  |
| renal replacement therapy |  |  | 7.077 | 0.800 |
| No | 20(40.0%) | 27(20.6%) |  |  |
| Yes | 30(60.0%) | 104(79.4%) |  |  |
| BMI, kg/m^2^ | 23.04 (21.13,25.29) | 22.29 (19.61,24.36) | 1.758 | 0.079 |
| SBP, mmHg | 148.45±18.25 | 143.99±17.50 | 1.184 | 0.237 |
| DBP, mmHg | 86.74±14.16 | 79.64±14.58 | 2.951 | 0.404 |
| Scr, mg/dl | 7.34  (6.16,9.96) | 8.88 (7.21,10.86) | -2.938 | 0.300 |
| BUN, mmol/L | 20.22±3.99 | 23.87±4.48 | -5.052 | ＜0.001 |
| eGFR，min·1.73m^2 | 8.55 (6.03,10.69) | 6.46 (5.28,8.45) | 3.81 | 0.301 |
| SUA，umol/L | 442.79±79.28 | 480.77±108.92 | -2.583 | ＜0.001 |
| P, mmol/L | 1.17±0.11 | 1.44±0.11 | -14.982 | ＜0.001 |
| Ca, mmol/L | 2.23±0.25 | 2.14±0.25 | 3.263 | 0.101 |
| iPTH，pg/Ml | 247.59 (201.23,369.83) | 248.03 (186.7,401.62) | 0.117 | 0.907 |
| ALP, U/L | 68.5 (47.35,89.75) | 73 (46.15,98) | -0.34 | 0.734 |
| TC, mmol/L | 4.38 (3.76,6.01) | 4.33 (3.67,5.3) | 0.934 | 0.350 |
| TG，mmol/L | 1.94±0.64 | 1.73±0.47 | 2.097 | 0.040 |
| HDL-C，mmol/L | 1.01 (0.85,1.23) | 0.96 (0.84,1.15) | 0.996 | 0.319 |
| LDL-C，mmol/L | 2.4 (1.96,3.39) | 2.4 (1.85,3.04) | 0.885 | 0.376 |
| HGB, g/dl | 106.39±16.62 | 99.96±21.45 | 2.139 | 0.335 |
| ALB，g/L | 36.95±5.76 | 36.82±6.75 | 0.117 | 0.907 |
|  |  |  |  |  |

Supplementary Table S3 Baseline characteristics of patients grouped by blood uric acid level

| Dependent | normal uric acid group(n=167) | hyperuric acid group(n=377) | t/χ2 | *P*-value |
| --- | --- | --- | --- | --- |
|  |  |  |  |  |
| Age | 48.26±13.72 | 62.63±13.89 | -11.171 | <0.001 |
| Gender |  |  | 1.759 | <0.001 |
| Female | 85(50.9%) | 215(57.0%) |  |  |
| Male | 82(49.1%) | 162(43.0%) |  |  |
| Smoking |  |  | 5.800 | 0.016 |
| No | 128(76.6%) | 321(85.1%) |  |  |
| Yes | 39(23.4%) | 56(14.9%) |  |  |
| Drinking |  |  | 0.058 | 0.810 |
| No | 153(91.6%) | 343(91.0%) |  |  |
| Yes | 14(8.4%) | 34(9.0%) |  |  |
| Diabetic |  |  | 17.934 | <0.001 |
| No | 125(74.9%) | 210(55.7%) |  |  |
| Yes | 42(25.1%) | 167(44.3%) |  |  |
| renal replacement therapy |  |  | 5.398 | 0.020 |
| No | 50(44.6%) | 142(32.9%) |  |  |
| Yes | 62(55.4%) | 290(67.1%) |  |  |
| BMI, kg/m^2^ | 23.14±4.38 | 22.71±3.54 | 1.111 | 0.267 |
| SBP, mmHg | 141.92±25.11 | 145.52±26.16 | -1.499 | 0.135 |
| DBP, mmHg | 84.35±15.58 | 78.76±14.44 | 4.063 | <0.001 |
| Scr, mg/dl | 7.95±2.15 | 9.05±2.69 | -5.083 | <0.001 |
| BUN, mmol/L | 15.64±7.60 | 22.08±8.28 | -8.573 | <0.001 |
| eGFR，min·1.73m^2 | 8.54±2.64 | 7.10±2.51 | 6.091 | <0.001 |
| P, mmol/L | 1.23±0.15 | 1.44±0.10 | -16.448 | <0.001 |
| Ca, mmol/L | 2.18±0.27 | 2.13±0.22 | 2.020 | 0.044 |
| iPTH，pg/Ml | 271.35±120.61 | 317.51±162 | -3.687 | <0.001 |
| ALP, U/L | 91.64±97.46 | 96.62±122.31 | -0.506 | 0.613 |
| TC, mg/dl | 4.96±1.68 | 4.51±1.27 | 3.092 | 0.002 |
| TG，mg/dl | 2.01±1.40 | 1.72±0.86 | 2.527 | 0.012 |
| HDL-C，mg/dl | 1.11±0.34 | 1.03±0.31 | 2.774 | 0.006 |
| LDL-C，mg/dl | 2.76±1.16 | 2.45±0.84 | 3.118 | 0.002 |
| HGB, g/dl | 115.36±21.89 | 101.35±21.62 | 6.948 | <0.001 |
| ALB，g/L | 38.56±6.34 | 36.74±6.58 | 3.016 | 0.003 |
| Complication |  |  | 1.604 | 0.658 |
| Hypertension | 56(50.0%) | 234(54.2%) |  |  |
| Coronary heart disease | 19(17.0%) | 74(17.1%) |  |  |
| Cerebral infarction | 9(8.0%) | 39(9.0%) |  |  |
